# Malaria and Malnutrition: Kwashiorkor Associated with Low Levels of Parasitaemia

**DOI:** 10.1155/2018/7153173

**Published:** 2018-09-27

**Authors:** Per Fevang, Kirsten Havemann, Børre Fevang, Arne T. Høstmark

**Affiliations:** ^1^Research Institute of Internal Medicine, Oslo University Hospital, Oslo, Norway; ^2^Danish Ministry of Foreign Affairs, Copenhagen, Denmark; ^3^Department of Community Medicine and Global Health, Institute of Health and Society, University of Oslo, Oslo, Norway

## Abstract

**Background:**

The relationship between protein energy malnutrition (PEM) and malaria is controversial. While most studies demonstrate that PEM is associated with greater malaria morbidity, some indicate that PEM may in fact have a protective effect. PEM is differentiated into three subgroups: kwashiorkor (marked protein deficiency), marasmus (calorie deficiency), and kwashiorkor/marasmus. None of the studies concerning PEM and malaria seem to distinguish between these subgroups, and significant differences in susceptibility to malaria between these subgroups may have been overlooked. Plasmodium parasites and malaria infected erythrocytes are sensitive to oxidative stress. Since kwashiorkor patients seem to display an excess of prooxidants and as serum albumin is an important antioxidant, we hypothesized that patients with different forms of PEM might have different levels of malaria parasitaemia.

**Methods:**

72 PEM children older than 6 months admitted to Kwale Family Life Training Programme (Kenya) were included in the study.

**Results:**

Mean parasitaemia was significantly lower in the kwashiorkor group than in the marasmus group (p < 0,001). There was no correlation between serum albumin and parasitaemia.

**Conclusion:**

Our study suggests a protective effect of kwashiorkor against malaria, warranting further studies.

## 1. Introduction

The relationship between protein energy malnutrition (PEM) and malaria is controversial. In a review concerning malaria and nutrition most studies demonstrated that PEM is associated with greater malaria morbidity and mortality; on the other hand some studies indicated that PEM may protect against malaria [1–3]. There are reports that malnutrition itself could modulate susceptibility to the disease [4]. Furthermore, there are reports of malaria exacerbation during refeeding of malnourished patients [5, 6].

PEM is differentiated into three subgroups:

1. Kwashiorkor (K): Protein malnutrition dominant, grossly hypoalbuminaemic with oedemas, and serum albumin <40 g/l.
2. Marasmus (M): Deficiency in calorie intake, hypo- or normoalbuminaemic.
3. Marasmus–Kwashiorkor (K/M): Marked protein deficiency (serum albumin < 40 g/l) and marked calorie insufficiency signs present, sometimes referred to as the most severe form of malnutrition.

As far as we know none of the studies concerning PEM and malaria distinguish between these three subgroups, and thus significant differences in susceptibility to malaria between these subgroups may have been overlooked.

In this study, based on previous experiments in mice and* in vitro,* we hypothesized that patients with low levels of albumin as seen in kwashiorkor would have lower levels of parasitaemia as compared to other patients with malnutrition [7–9]. Malnourished children living in an endemic area of* P falciparum* infection were tested for parasitaemia and classified according to PEM subgroup.

## 2. Subjects and Methods

### 2.1. Patients

Patients older than 6 months admitted to Kwale Family Life Training Programme [FLTP] in the coastal province of Kenya were invited to participate in the study consecutively and included after informed oral consent from the mother. Patients were assigned to clinical subgroups based on clinical evaluation and appearance including oedema, weight, weight/length, and weight/age.

Exclusion criteria were treatment with antimalarial drug within 1 week before admittance, fever above 37.5°C and hospitalisation or treatment for malaria on the day of admittance, or during the stay. Other criteria for exclusion during the stay were vomiting more than 2-3 times a day and/or diarrhoea more than 5 times a day for more than 2 days during the stay.

Seventy-two malnourished children were included in the study (Table 1). Sex, age, body weight, length, and medical and social history were recorded. Haemoglobin, stool microscopy, and urine microscopy were performed, thick and thin bloods smears were made, and blood samples were obtained for the determination of serum albumin. Blood samples for serum albumin were unfortunately not obtained from all patients due to limited capacity for venous blood sampling among staff. There was no systematic selection of patients for venous blood sampling.

Seventeen patients were assigned to the K group based on the presence of pitting oedemas, distended abdomen, and typical dermatitis with normal body weight, and blood for determination of serum albumin was obtained from ten patients.

Forty-five patients were assigned to the M group based on a body weight of less than 60% of the expected weight for age, and blood for determination of serum albumin was obtained from thirty of them.

Ten patients were assigned to the K/M group based on the presence of symptoms of both kwashiorkor and marasmus, and serum albumin was obtained from nine of them.

### 2.2. Ethics

The study was performed according to the Declaration of Helsinki and was approved by the ethical committee of the provincial health authority. After adequate explanation of the aim of the study informed consent was obtained from the parents of the children.

### 2.3. Analysis

The blood smears were stained with a 5% Giemsa solution and the parasites [asexual forms of* P. falciparum*] were counted by experienced laboratory personnel against 1000 WBC and converted to parasites pr microliter blood assuming 8000 WBC/microliter. The analysis of serum albumin was performed at Aalborg Hospital, Denmark by an immunochemical method [Behring Nephelometer Analyzer, Behringwerke, Germany].

### 2.4. Statistical Analysis

Differences between groups were tested by the Kruskal–Wallis and Mann–Whitney tests as appropriate. Correlation was tested using the Spearman test. Levels of parasitaemia were adjusted for the effect of age using linear regression. A p-value below 0,05 was considered significant.

## 3. Results

### 3.1. Albumin

**Figure 1:**
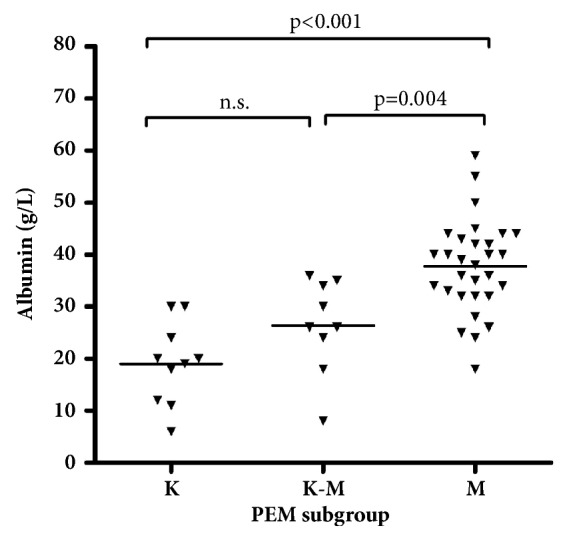
Levels of serum albumin in three subgroups of protein energy malnutrition (PEM); kwashiorkor (K), kwashiorkor-marasmus (K-M), and marasmus (M). Levels compared using the Kruskal–Wallis and Mann–Whitney method.

Serum albumin concentration was determined from the majority of the PEM children; i.e., in 10 with K, 9 with K/M, and 30 with M. Mean serum albumin was significantly lower in the K and K/M group than in the M group (PEM p <0.001 and p=0.004, respectively) supporting the clinical evaluation of the children (Figure 1).

### 3.2. Parasitaemia

**Figure 2:**
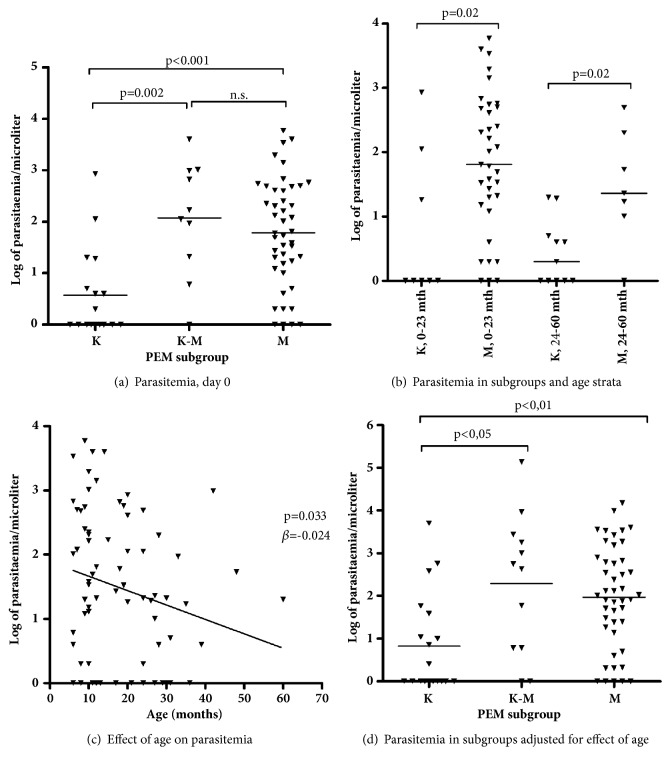
(a) Levels of parasitaemia in the three subgroups of PEM. (b) Parasitaemia in children age 0-23 and 24-60 months in the kwashiorkor [K] and marasmus [M] subgroup. (c) Correlation of parasitaemia and age in children of all subgroups. Line depicting slope of linear regression. (d) Parasitaemia in the tree subgroups adjusted for the effect of age. Levels compared using the Kruskal–Wallis and Mann–Whitney method (panels a, b, and d).

Mean parasitaemia was significantly lower in the clinically assessed K group than in the K/M and M group, when all patients were combined (p = 0,002 and p<0.001, respectively) (Figure 2(a)). The differences in age between the three subgroups (Table 1) could potentially have influence on this finding, but the finding was consistent even when dividing the patient population in two different age strata (0-23 and 24-60 months, respectively) (Figure 2(b)), even if there was a weak correlation between age and parasitaemia (Figure 2(c)). Moreover, adjusting the level of parasitaemia for the effect of age did not alter the main finding, showing significantly lower levels of parasitaemia in the K group compared to the K/M and M group (Figure 2(d)).

There was no significant correlation between serum albumin and parasitaemia (data not shown).

## 4. Discussion

In our study, we found significantly lower levels of* P falciparum* parasitaemia in malnourished children clinically assessed as kwashiorkor as compared to kwashiorkor/marasmus and marasmus, indicating an association between the different groups of PEM and levels of parasitaemia.

The malaria parasite needs 48-72 hours in an intact erythrocyte host cell to survive and mature and a tenuous balance exists between the requirements for successful replication of the malaria parasite and the maintenance of an intact host erythrocyte [10]. It is reported that malaria infected red cells have a reduced defence against oxidation and thus would be vulnerable to premature haemolysis leading to parasite death in a situation of oxidative stress [11].

In kwashiorkor, there seems to be an excess of prooxidants leading to an excess in free radicals. The activity of the antioxidative enzyme glutathione peroxidase, as well as the concentration of its substrate, and reduced glutathione, was significantly lower in serum of kwashiorkor children than in marasmic children and in healthy children [12]. It has further been demonstrated that the concentration of free fatty acids is increased in kwashiorkor [13] and free fatty acids in the erythrocyte membrane are prone to peroxidation.

In vitro, the peroxide-prone fatty acids of fish oil have been shown to greatly inhibit growth of* P. falciparum*, while the antioxidant vitamin E worsened the infection [8, 14]. Likewise, studies in mice demonstrated an antiparasitic effect of a diet of fish oil [7, 15], while the antioxidant vitamin E reversed the suppressive action of the fish oil diet. It has also been demonstrated that the antiparasitic effect of fish oil has a long-term protection against malaria in mice [7].

Albumin is an important extra cellular antioxidant [16], covering 40% of the antioxidant capacity in the serum of healthy persons [17]. In cell cultures albumin strongly prevented peroxidation of added PUFAs [9]. Moreover, albumin counteracts the haemolytic effect of polyunsaturated fatty acids [18]. It has also been shown* in vitro* that parasite growth increased with increasing concentration of albumin up to 10 g/l [8]. In our study, we did not find an association between serum albumin and parasitaemia, so the clinical significance of the in vitro studies is uncertain. A protective effect of kwashiorkor could therefore be mediated through other proteins with antioxidant properties.

Patients with the sickle cell trait are partially protected against malaria, but the mechanism underlying this paradox phenomenon is still under debate [19, 20]. The content of the important antioxidant vitamin E in sickle cell trait erythrocytes are considerably lower than normal and sickle cell trait erythrocytes have increased susceptibility to oxidation [21]. Recent studies suggest that several mechanisms contribute to the high oxidative burden in sickle cell patients [22, 23]. Hypothetically, some of the protective effects of both famine and sickle cell trait in malaria might be attributed to increased premature haemolysis of infected erythrocytes due to oxidative stress. In keeping with this hypothesis, increased osmotic fragility of human erythrocytes exposed to peroxidation prone fatty acids in vitro was observed [18].

There is no general agreement about the rate of onset of acquired immunity or what constitutes the key determinants of protection, and there seems to be no consensus regarding the mechanism[s] of protection [24]. Age is a significant risk factor for malaria prevalence and parasite density, but age does not appear to influence the incidence of the disease [25]. In our study, there was no association between age and parasitaemia. A major limit to our study is the small number of participants and the lack of other markers of oxidative stress and circulating levels of oxidants and antioxidants.

In summary, our study indicates a difference between subgroups of protein energy malnutrition (PEM) concerning malaria parasitaemia and suggests a protective effect of kwashiorkor. As far as we know, previous studies have not distinguished between kwashiorkor and marasmus in PEM and more studies are needed to elucidate our hypothesis.

## Figures and Tables

**Table 1:** 

	Kwashiorkor	Kwashiorkor/marasmus	Marasmus	P-value
Sex (male/female)	8/9	7/3	13/32	
Hemoglobin (g/dL)	8.0 (4.0-9.0)	7.0 (4.0-10.0)	6.0 (4.0-10.0)	<0.01
Temperature (C)	36.3 (35.5-37-4)	36.6 (36.3-36.9)	36.4 (34.4-38.7)	0.59
Weight (kg)	7.3 (6.6-10.5)	6.6 (4.9-8.6)	5.2 (3.6-8.3)	<0.01
Age (months)	24 (8-60)	18 (10-42)	10 (6-48)	<0.01
Weight/length	12 (8.2-18.9)	11 (8.9-15.5)	8.9 (7.5-16.7)	<0.01

Values are median and range with exception of ^*∗*^. ^*∗*^Numbers of each sex.

## Data Availability

Data of the study is available as a supplemental excel file.
